# Disability in subjects who survive road traffic injuries in 2 regions of southwest Colombia: A causal mediation analysis

**DOI:** 10.1016/j.cjtee.2025.01.005

**Published:** 2025-07-23

**Authors:** Lina Marcela Sandoval, Andrés Fandiño-Losada, Elvis Siprian Castro-Alzate, Claudio Bustos, Alberto Federico García, Adrián David Fernández

**Affiliations:** a CISALVA Institute Universidad del Valle, Cali, Colombia; b CISALVA Institute, Faculty of Health.Universidad del Valle, Cali, Colombia; c SINERGIA Grupe. Faculty of Health. Universidad del Valle, Cali, Colombia; d Department of Psychology, Faculty of Social Sciences, Universidad de Concepción, Concepción, Chile; e Fundación Clínica Valle del Lili, Hospital Universitario del Valle, Universidad del Valle, Universidad, ICESI, Cali, Colombia; f QUIRON SALUD Grupe, Clínica Imbanaco, Universidad del Valle, Cali, Colombia

**Keywords:** Disability, Mediation analysis, Traffic accidents

## Abstract

**Purpose:**

The objective of the study was to determine the causal interrelationships between sociodemography, clinic, and injury characteristics, and to access to rehabilitation services that generate disability in road traffic injury survivors in 2 regions of Southwest Colombia during the 2018–2021 period.

**Methods:**

An ambispective cohort study included 261 survivors from road traffic in 2 regions of Southwest Colombia (Cauca and Valle del Cauca) between April 19, 2021 and June 03, 2022. These survivors accepted treatment in 3 high-level comprehensive health institutions in Cali, Colombia, which are regional referral centers for trauma in the region. Patients (1) with hospitalization time ≥12 h, (2) aged ≥18 years, and (3) having the ability to understand the research questionnaires by themselves or their legal representatives, were included. Those with a history of deficiency before the road traffic injury were excluded. A structural equation model of causal pathways of disability was established to estimate exposure variables that are sociodemography, clinic, and access to rehabilitation services. The main outcome variable was disability, estimated by the World Health Organization Disability Assessment Schedule 2.0. Exposure variables related to clinical characteristics were measured through medical record review. The variables of access to rehabilitation services and disability were measured through a telephone-based survey. Structural equation analysis was performed, estimating the mediating effects of disability. The analyses were conducted in the software R Studio® y Mplus®.

**Results:**

The study found a significant gender difference in disability, with women presenting a significantly higher disability than men (β: 6.21; *p* = 0.041). Disability was also associated with clinical conditions, such as injury severity score (β: 0.67; *p* < 0.001) and length of hospitalization (β: 0.28; *p* < 0.001). Regarding access to health services, the risk of disability was higher among those who were readmitted to the health institution (β: 4.96; *p* < 0.001).

**Conclusions:**

Disability caused by road traffic injuries must be conceived as a complex phenomenon to be studied, involving the non-linear interaction between the individual's deficiencies and contextual factors.

## 1 Introduction

Among external injuries, road traffic injuries are the leading cause of death in the world.^1^ In addition, these injuries generate an enormous economic burden for health care services and victims, and lead to psychological,^2^^,^^3^ social,^4^ and occupational^5^^.^^6^ consequences, becoming one of the main causes of disability in people of productive age.^7^ Road traffic injuries and their consequences are also a problem affecting the Colombian population in different age groups.^8^^,^^9^ Southwest Colombia concentrates one-sixth of the national population. Buenaventura, as a major agro-industrial center on the Pacific Ocean, experiences high freight and passenger land transport flows. Additionally, Valle del Cauca is the main region of this area and contributes the highest number of deaths due to road traffic injuries in Colombia for 2020, with 760 cases. It is also one of the 5 regions that reported the highest number of injuries due to road traffic for the same year, after Antioquia and Bogotá.^10^

Furthermore, under the biopsychosocial model, disability defined by the International Classification of Functioning, Disability and Health emerges as a process of continuous adjustment between the individual's capacity with a specific health condition and external factors, including their living circumstances, environmental demands, and societal expectations.^11^ Thus, disability is a generic term that encompasses the presence of impairments, activity limitations, and participation restrictions, which implies the existence of an interaction between the individual and the environment.^11^ Under this approach, disability is understood from a complex structure establishing interdependence between the individual characteristics and context.^11^

Research conducted in other countries,^3^^,^^12^, ^13^, ^14^, ^15^, ^16^ have evaluated disability in trauma survivors through World Health Organization Disability Assessment Schedule, 2002 (WHODAS 2.0) that assesses functioning and disability,^17^ reporting disability values of 55.8%,^14^ 42%,^12^ and 63%,^12^ and with factors such as the level of severity of the injury,^3.13^ hospital stay,^14^ location of the injury in the extremities,^3.13^ and Glasgow at discharge^16^ as associated with the level of disability installed. Two studies conducted in Norway and New Zealand evaluate the condition of access to health services and their relationship with disability in trauma victims.^15^^,^^18^ However, the interrelationships between access to rehabilitation services and sociodemographic and clinical conditions as explanatory factors of disability in traffic injury survivors are unknown.

The study aims to evaluate the causal interrelationships between sociodemography, clinic characteristics, and conditions of access to rehabilitation services for disability level in road traffic survivors between 2018 and 2021 in Southwest Colombia, and to evaluate the mediating role of access to rehabilitation services in the relationship between disability level and clinical characteristics of trauma.

## 2 Methods

An institutionally based ambispective (i.e., retrospective and prospective) cohort study was conducted as an approach to infer causality when random assignment of exposure is not possible.^19^ Thus, the exposure variables of the sociodemographic and clinical components were first identified, and subsequently the characteristics of access to rehabilitation services and the disability.

During 2018 to 2021, traffic injury survivors from 2 regions of Southwest Colombia (Cauca and Valle del Cauca) who were treated in 3 high-level comprehensive health institutions in Cali, Colombia, which are regional referral centers for trauma in the area, were included in the study. Inclusion criteria were: (1) hospitalization time ≥12 h, (2) aged ≥18 years, (3) survivors and their legal representative can understand the research questionnaires. Those with a history of deficiency before the road traffic injury were excluded. For the sample size calculation, disability was used as the outcome variable. Participants with an injury severity greater than 2.0 on the abbreviated injury scale^20^ were classified as exposed. The calculation assumed a relative risk of 1.42, a 95% confidence level, 80% statistical power, and a 46% incidence of disability among non-exposed subjects,^21^ based on data from Derrett et al.^18^ Therefore, the calculated sample size was 245 subjects, adjusting for a 20% non-response rate.

### 2.1 Instruments

Exposure variables related to clinical characteristics, such as hospital inpatient stay times, intensive care hospitalization, and injury severity score (ISS), were measured through medical record review. The variables of access to rehabilitation services and disability were measured through a telephone-based survey. To measure the conditions of access to rehabilitation services, an instrument was designed, which was subjected to content validation by experts. Disability was evaluated with the 36-item version of the World Health Organization Disability Assessment Schedule, 2000 (WHODAS 2.0), which has a test-retest reliability level of 0.98 at a general level^22^ and is validated in trauma patients and for telephone administration.^23^^,^^24^

The research was approved by the Human Ethics Committee of the Universidad del Valle according to records id 015–020 and by the Ethics Committee of the participating institutions according to records of 2020 with the id 049 from Hospital Universitario del Valle, 000120 from Clinica Imbanaco, and 386 from Fundación Valle del Lili.

Data collection was obtained after the year in which the road traffic injury occurred and consisted of the following sequential activities: (1) reviewing of the databases consisting the subjects admitted for road traffic injuries provided by the institutions during the study period; (2) verifying inclusion criteria, confirmation and registration of contact telephone numbers; (3) contacting and recruiting subjects by telephone in the study. The telephone-based survey, at this first stage, lasted an average of 40 min.

### 2.2 Statistical analysis

A structural equation model (SEM) of disability was estimated. For this purpose, a theoretical model was determined, formed by the disability constructed as the main endogenous variable of the model and 2 groups of measured variables: (1) clinics, where the ISS and hospitalization time in intensive care unit (ICU) and in general and emergency rooms are the endogenous variables; (2) variables of access to rehabilitation services, where not receiving rehabilitation is the endogenous variable. The sociodemographic variables and the presence of antecedents were considered together with injury duration and follow-up time as the exogenous variables of the model, and possible causal pathways of disability among these groups of variables were raised. Subsequently, multiple regression models were estimated for each defined endogenous variable to establish the explanatory variables of disability that conform to the SEM model of causal pathways of disability. The models were determined in successive steps, as follows: (1) Multiple linear regression models were estimated for ISS, hospitalization in inpatient and emergency rooms, and disability. These variables were analyzed as continuous variables with robust estimator of variance in both the previous steps and the final model. (2) A logistic regression model was estimated for the variable not receiving rehabilitation.

The variable selection process for the final multiple models was performed by the backward method using the Akaike criterion^25^ as an adjustment indicator. The final models included variables with statistical significance (*p* < 0.20) and those deemed theoretically important.^26^ As for the regression of the disability score, the collinearity between the access variables was evaluated through Spearman's correlation coefficient, and the interaction between clinical, sociodemographic, and access variables was evaluated through the inclusion of this term in the models. These analyses were performed in the R Studio® statistical software. The disability causal pathways model was estimated through the SEM analysis, formed by the previously defined regression models.^26^^,^^27^

Additionally, during the model estimation, 2 variables were identified with *p* values below 0.01 in the causal pathway of disability: presence of a caregiver and hospitalization after hospital discharge, so they presented as endogenous variables in the model related to access to rehabilitation services. The SEM model was estimated using the Weighted Least Squares Means and Variance Adjusted estimator. Considering that the sample size was relatively small, a free-form estimation and the non-normal distribution of endogenous variables were used, and the missing data were addressed through the expectation-maximization algorithm, which calculates the best possible estimator.^27^^,^^28^ In addition, modification rates were calculated, and the goodness of fit was evaluated through the comparative fix index (CFI), the Tucker-Lewis index (TLI), and the root mean square error of approximation (RMSEA). A good model fit was considered when the CFI and TLI were greater than 0.98 and the RMSEA was less than 0.05.^29^^,^^30^

The determination of the mediating effects of disability was performed considering the following fundamental equation of mediation analysis:^31^

c = ab + c0

The total effects of the clinical and sociodemographic variables on disability (*c*), which are the unconditional effects of these on disability, were estimated. For the mediation analysis, the total effect was decomposed into 2 parts: (1) *ab* corresponds to the effect of clinical and sociodemographic variables mediated by access conditions to generate disability: mediated or indirect effects. (2) *c0* corresponds to the effect of sociodemographic and clinical variables not mediated by access conditions, called direct effects.^31^ Subsequently, variables with significant direct, indirect, and total effects on disability were identified. Specific indirect effects (mediating effects) of ISS on disability were evaluated. Finally, the confidence intervals were calculated using the Bootstrap method, which allows for obtaining resamples from the original sample *ab* and calculating the confidence interval based on the distribution of these *ab*. SEM analyses were performed in Mplus‍ version 8.5®.^31^

## 3 Results

**Fig. 1:**
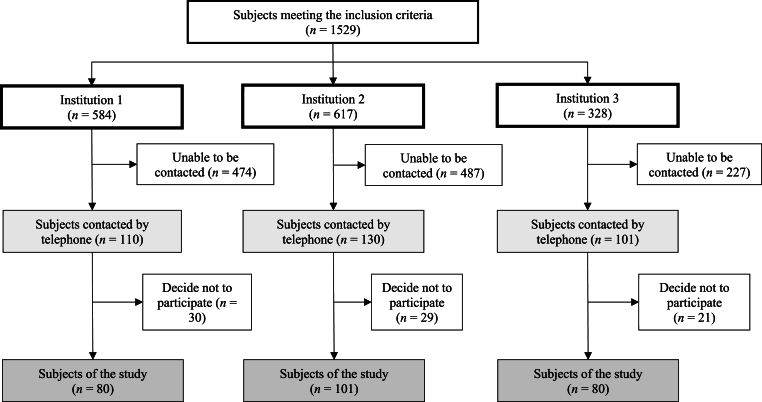
Recruitment of subjects throughout the study.

Data collection began on April 19, 2021 and ended on June 03, 2022. In total, 1529 subjects met the inclusion criteria and were available to be contacted, of whom 30.2% (*n* = 584) were attended at Hospital Universitario del Valle (institution 1), 40.4% (*n* = 617) at Clínica Imbanaco (institution 2), and 21.5% (*n* = 328) at Fundación Valle del Lili (institution 3). There were 341 subjects answered the calls and were invited to participate, 76.5% (*n* = 261) of whom accepted to participate and became participants in the study (Fig. 1). The median time from road traffic injury to participant contact was 20.40 months (12.36, 29.51).

The comparison between participants and non-participants according to the treatment institution, sex, ICU requirement, affected body area, age, and hospitalization time was performed (Table 1). There were differences between participants and non-participants according to sex (*p* = 0.001). Of the non-participants who met the inclusion criteria, 71.7% (*n* = 1109) were men and 28.3% (*n* = 438) were women. In contrast, among the participants, 62.1% (*n* = 162) were men and 37.9% (*n* = 99) were women (*p* = 0.011). There were no statistically significant differences between participants and non-participants in terms of treatment institution, ICU requirement, and length of hospitalization (Table 1).

**Table 1:** Participant and non-participant characteristics.

Variables	Non-participant (*n* = 1547)	Participant (*n* = 261)	*p* value
Institutions			0.231Ω
Institution 2	516 (33.4)	101 (38.7)	
Institution 1	504 (32.6)	80 (30.7)	
Institution 3	527 (34.1)	80 (30.7)	
Sex			0.002Ω
Women	438 (28.3)	99 (37.9)	
Men	1109 (71.7)	162 (62.1)	
ICU requirement, *n* = 1303^a^			0.071Ω
Yes	296 (28.4)	59 (22.6)	
No	746 (71.6)	202 (77.4)	
Age (year)	34 (26 – 49)	34 (26 – 47)	0.672∗
Hospitalization time (day), *n* = 1494^b^	4 (1 – 9)	3 (2 – 9)	0.973∗

Data presented as *n* (%) or median (Q_1_, Q_3_).

Ω: Chi-square; ∗: Mann-Whitney; ICU: intensive care unit.

a “ICU requirement” was not reported in the database provided by all institutions.

b “Hospitalization time” was not reported in the database provided by all institutions.

The median age of the subjects was 34.0 (26–47) years, of which 38.5% (*n* = 101) were aged 31 – 50 years, and 62.1% (*n* = 162) were men. A majority (52.9%, *n* = 138) reported having no partner, while 36.7% (*n* = 95) had a high school as the highest educational level. The most frequent socioeconomic level among the participants was 2 (low), representing 36.0% (*n* = 90) of the study. Regarding the health affiliation regime, 66.3% (*n* = 171) were enrolled in the contributory regime, while 85.0% (*n* = 217) reported being employed as their main occupation before the road traffic event.

Regarding the clinical characteristics, 78.1% (*n* = 204) of the subjects had no significant medical history at the time of the event. Injuries with an abbreviated injury scale > 2 in the extremities were observed in 33.3% (*n* = 86) of cases. ICU admission was required for 22.6% (*n* = 59), and 77.4% (*n* = 202) underwent at least 1 surgery during hospitalization. The median ISS of the subjects was 9.0 and a quarter presented values > 10 (4.0, 10.0). Additionally, the largest proportion 46.3% (*n* = 119), presented an ISS < 9, while 5.1% presented an ISS ≥ 25. The median time in the inpatient and emergency room was 3.0 days (1.0, 7.0), while the median time in the ICU for those requiring this service (*n* = 59) was 4.0 days (2.0, 8.7). Regarding access to rehabilitation services, 86.9% (*n* = 227) of the subjects reported having no caregiver for daily activities. Additionally, 30.2% (*n* = 77) reported affected health condition (level 4: very affected) after the road traffic event, and 57.8% (*n* = 111) rated the impact of rehabilitation services on their recovery as level 4 (highly influential).

The proportion of subjects who received rehabilitation was 74.7% (*n* = 195). Following hospital discharge, 70.7% (*n* = 183) reported not requiring surgery, and 83.4% (*n* = 216) were not readmitted. Of those receiving rehabilitation, 43.3% received at least one rehabilitation service, with a median duration of 275.5 days (120.1, 609.2). Of the 195 subjects who received rehabilitation, 52.1% (*n* = 100) rated the service quality as medium on the assessment scale. Regarding costs, 45.1% (*n* = 88) reported the Mandatory Insurance Against Traffic Accidents as their exclusive payment source for rehabilitation services.

The median disability of the study subjects was 14.9 (3.8, 97.6). According to disability categories defined by the International Classification of Functioning, Disability and Health (CIF-IA),^11^ the majority of participants presented with mild disability 36.5% (*n* = 96), while only 1 (0.4%) participant presented extreme disability (Table 2).

**Table 2:** Disability levels in study subjects (*n* = 261).

Disability	*n* (%)
0 – 4: None	68 (26.2)
5 – 24: Mild	96 (36.5)
25 – 49: Moderate	57 (21.9)
50 – 95: Severe	39 (15.0)
96 – 100: Extreme	1 (0.4)

The bivariate analysis between the main sociodemographic, clinical, and access to rehabilitation services characteristics and the disability levels was performed. The median disability score was significantly higher among: (1) participants with only primary school education, (2) women, (3) those with greater injury severity (ISS > 25), (4) ICU admission, and (5) non-ICU ward patients with hospital stays ≥ 3 days (Table 3).

**Table 3:** Disability and main socio-demographic, clinical, and access to rehabilitation services characteristics, median (Q_1_, Q_3_).

Characteristic	Disability	*p* value
Sociodemographic		
Age (year)		0.892
18–30	13.03 (3.60, 37.23)	
31–50	15.47 (3.17, 35.19)	
>50	16.79 (4.61, 39.29)	
Sex		0.001^a^
Women	24.44 (5.60, 41.68)	
Men	12.79 (2.38, 30.35)	
Marital status		0.523^a^
With partner	18.50 (2.99, 36.45)	
Without partner	12.79 (3.89, 36.05)	
Region of residence		0.792^a^
Valle del Cauca	14.58 (3.39, 36.97)	
Cauca	21.72 (9.28, 23.69)	
Maximum educational level		0.001^b^
Elementary	31.39 (14.54, 57.17)	
High school	17.61 (5.90, 41.87)	
Technician	11.96 (4.16, 33.39)	
Bachelor's degree	6.10 (1.56, 24.45)	
Socioeconomic level (*n* = 250)		0.123^b^
0–1	21.48 (5.97, 46.73)	
2	16.25 (2.97, 34.72)	
3	14.86 (4.46, 39.04)	
>3	7.67 (2.09, 30.68)	
Health affiliation regime (*n* = 258)		0.092^a^
Contributory	11.25 (2.14, 33.80)	
Subsidized	13.15 (3.75, 36.94)	
Clinics		
Medical history		0.273^b^
None	14.10 (2.92, 36.02)	
HTN and/or DM	23.18 (10.84, 38.70)	
Other	17.29 (4.70, 35.74)	
ISS		0.012^b^
<9	9.72 (1.63, 29.84)	
9–15	19.94 (16.84, 36.16)	
16–24	29.20 (5.43, 43.02)	
≥25	49.16 (15.35, 63.05)	
ICU requirement		0.031^a^
Yes	29.44 (3.31, 55.19)	
No	12.88 (3.77, 33.30)	
Surgery requirement		0.801^a^
Yes	14.58 (4.16, 36.07)	
No	14.93 (2.08, 36.97)	
Days of hospitalization in ICU (*n* = 59)		0.072^a^
1–3.99	20.69 (1.14, 40.14)	
4–49	37.55 (10.16, 58.31)	
Days of hospitalization in inpatient and emergency rooms		0.011^a^
0.5–2.99	8.60 (2.08, 24.31)	
3–72	21.60 (6.11, 45.40)	
Access to rehabilitation services		
Presence of a caregiver		0.011^a^
Yes	48.80 (28.19, 62.87)	
No	11.87 (2.82, 32.22)	
Have received or receive rehabilitation		0.032^a^
Yes	18.33 (4.49, 39.38)	
No	8.32 (2.20, 26.14)	
Post-discharge surgery (*n* = 259)^c^		0.011^a^
Yes	29.85 (8.37, 49.89)	
No	11.48 (2.26, 29.90)	
Post-discharge hospitalization (*n* = 259)^c^		0.011^a^
Yes	37.50 (16.84, 55.29)	
No	11.87 (2.75, 32.85)	

HTN: Hypertension; DM: Diabetes mellitus; ISS: injury severity score; ICU: intensive care unit.

a U de Mann Whitney.

b Kruskal Wallis.

c Indicate variable with 3 missing data.

### 3.1 SEM model of causal pathways of the level of disability

**Fig. 2:**
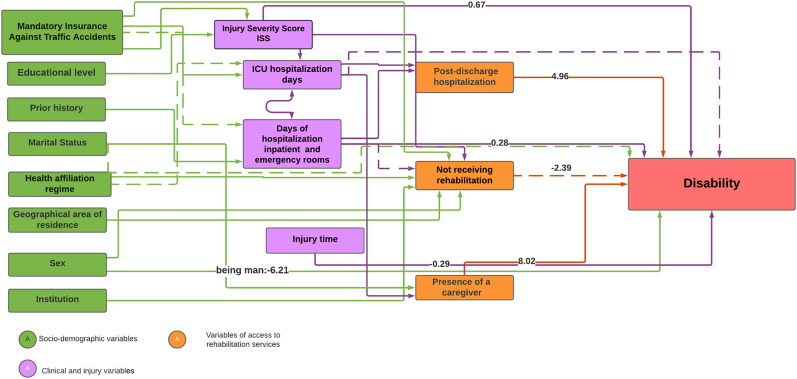
SEM model of causal pathways to disability. SEM model of causal pathways of disability time 1. Continuous arrows represent causal relationship significant at *p* < 0.100, dashed arrows represent relationship significant at *p* < 0.200. SEM: structural equation model; ISS: injury severity score; ICU: intensive care unit.

Table 4 and Fig. 2 present the results of the causal pathways model of disability. The model showed that disability among the study participants was associated with sex, with women presenting a higher risk of disability compared to men (β men: 6.21, *p* = 0.041). Disability was also associated with clinical conditions, such as injury severity score (β ISS: 0.67, *p* < 0.001) and length of hospitalization (β: 0.28, *p* < 0.001). Regarding access to health services, the risk of disability was higher among those who were readmitted to the health institution (β: 4.96; *p* < 0.001) and required a caregiver (β: 8.01; *p* < 0.001). The model presented an adequate adjustment to the data (Chi^2^: 103, *p* = 0.690, CFI: 1.0, TLI: 1.0, RMSEA: 0.00) and thus the theoretical model of causal pathways of disability fits the data.^29^

**Table 4:** SEM model of causal pathways of disability.

Variables	Disability	
	Coefficient	*p* value
Sex		0.041
Women	0	
Men	−6.21	
Age	0.03	0.782
Marital status		0.172
With Partner	0	
Without Partner	6.00	
Time of injury (months)	−0.29	0.021
Institution		
Institution 2	0	
Institution 3	−4.46	0.263
Institution 1	4.05	0.212
ISS	0.67	< 0.001∗
Days of hospitalization in ICU	−0.62	0.143
Days of hospitalization in inpatient and emergency rooms	0.28	< 0.001∗
Presence of a caregiver		< 0.001∗
No	0	
Yes	8.02	
Receiving rehabilitation		0.172
Yes	0	
No	−2.39	
Post-discharge hospitalization		< 0.001∗
No	0	
Yes	4.96	
Duration of rehabilitation (day)	0.01	0.011

SEM: structural equation model; ISS: injury severity score; ICU: intensive care unit; ∗: statistical significance.

### 3.2 Mediation analysis in the explanation of disability

Table 5 presents the direct, indirect, and total effects of sociodemographic and clinical conditions explaining disability. Male sex, ISS, and days of hospitalization (inpatient and emergency) presented a significant total effect on disability, while days of hospitalization in ICU presented a significant indirect effect.

**Table 5:** Direct, indirect, and total effects of sociodemographic and clinical characteristics on disability.

Variable	Coefficient	95% *CI*
Age		
Direct effect	0.03	−0.17 – 0.26
Indirect effect	0.01	−0.13 – 0.12
Total effect	0.03	−0.22 – 0.30
Sex (being man)		
Direct effect	−6.13	−12.04 – 0.20
Indirect effect	−1.82	−6.82 – 1.33
Total effect	−7.95	−13.61 – −2.94
Marital status (without partner)		
Direct effect	6.04	−0.99 – 37.13
Indirect effect	−5.57	−3.19 – 0.74
Total effect	0.47	−5.79 – 6.34
Institution 2		
Direct effect	4.04	-2-95 – 12.67
Indirect effect	−1.39	−6.96 – 2.81
Total effect	2.64	−5.20 – 11.31
Institution 3		
Direct effect	−4.34	−11.35 – 1.73
Indirect effect	−1.64	−5.85 – 2-72
Total effect	−5.98	−12.34 – 0.04
ISS		
Direct effect	0.68	0.03 – 1.50
Indirect effect	0.26	−0.83 – 1.02
Total effect	0.95	0.42 – 1.57
Days of hospitalization in ICU		
Direct effect	−0.78	−25.61 – 1.10
Indirect effect	1.42	0.57 – 24.07
Total effect	0.63	−0.28 – 3.90
Days of hospitalization in inpatient and emergency rooms		
Direct effect	0.28	−0.07 – 1.22
Indirect effect	0.14	−0.96 – 0.46
Total effect	0.42	0.01 – 0.86

ISS: injury severity score, ICU: intensive care unit, *CI*: confidence interval.

### 3.3 The relationship between ISS and disability

**Fig. 3:**
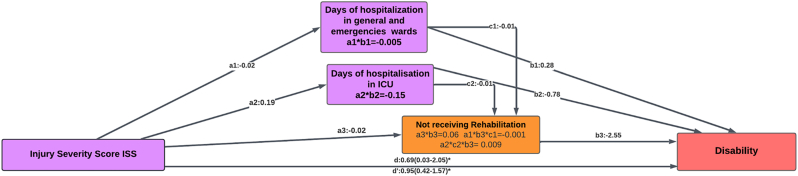
Pathway model of the mediation of not receiving rehabilitation between ISS and disability. ∗: Confidence interval of the coefficient does not include 0. ISS: injury severity score, ICU: intensive critical care.

Table 6 and Fig. 3 present the specific indirect effects, direct effect, and total effect of ISS on disability. According to the results, the direct effect (β: 0.69, 95% *CI*: 0.03–2.05) and the total effect (β: 0.95, 95% *CI*: 0.42–1.57) of ISS on disability are significant. It is observed that there is no evidence suggesting that the absence of rehabilitation mediates the relationship between the ISS and disability. Therefore, the indirect effect of ISS mediated by not receiving rehabilitation (indirect effect 1) was not significant (β: 0.06, 95% *CI*: 0.70–0.41). Similarly, indirect effects 5 and 6 mediated by not receiving rehabilitation combined with hospitalization were not significant. However, there is evidence of mediation of days of hospitalization in ICU/presence of a caregiver in the relationship between ISS and disability. For each 1-unit increase in ISS, disability increased by 0.25 per additional day in the ICU, and for each 1-unit increase in ISS, disability increased by 0.25 points when a caregiver was present after controlling for other mediators (indirect effect: 7, *ab*: 0.25, 95% *CI*: 0.10–3.43). No other mediating effects were identified in this relationship.

**Table 6:** Indirect, direct, and total effects of the relationship between ISS and disability.

Variables	Coefficient	95% *CI*
Direct effect	0.69	0.03–2.05
Indirect effect		
Not receiving rehabilitation	0.06	−0.70–0.41
Days of hospitalization in inpatient and emergency rooms	−0.00	−0.17–0.05
ICU hospitalization days	−0.15	−4.22–0.19
Re-enter the health institution	0.08	−0.89–0.34
Days of hospitalization in inpatient and emergency rooms (not receive rehabilitation)	−0.00	−0.02–0.03
ICU hospitalization days (not receive rehabilitation)	0.00	−0.13–0.44
ICU hospitalization days (with a caregiver)	0.25	0.10–3.43
Days of hospitalization in inpatient and emergency rooms (hospitalization after discharge)	−0.00	−0.04–0.04
ICU hospitalization days (hospitalization after discharge)	0.02	−0.16–0.84
Total indirect	0.26	−0.83–1.02
Total	0.95	0.42–1.57

ISS: injury severity score; ICU: intensive critical care; *CI*: confidence interval.

In Fig. 3, the coefficients *a1* to *a3* represent the coefficients of the regression between SSI and hospitalization in inpatient and emergency rooms, hospitalization in ICU, and receiving rehabilitation, respectively. Coefficients *b1* to *b3* represent the coefficients of the multiple regressions between hospitalization time, receiving rehabilitation, and disability time. The coefficients *c1* and *c2* represent the coefficients of the multiple regressions between days of hospitalization in inpatient and emergency rooms, days of hospitalization in ICU, and not receiving rehabilitation, respectively. Coefficient d is the direct effect of the ISS on disability, and d is the total effect of ISS on disability. The products *a3*∗*b3*, *a1*∗*b3*∗*c1*, *a2*∗*b3*∗*c2*, *a2*∗*b3*∗*c2*, represent the indirect effects of the relationship between ISS and disability mediated by not receiving rehabilitation.

## 4 Discussion

Injuries caused by external causes are an important public health problem in Latin America and the Caribbean. Although research on trauma and its care is extensive,^32^, ^33^, ^34^, ^35^, ^36^ the literature on disability in the region is scarcer.^37^ In addition, to our knowledge, no other study of trauma and disability has been previously conducted in the region through the analysis of causal pathways (path analysis). Therefore, the present study proposes an explanatory model of the disability in traffic injury survivors, from a biopsychosocial approach, thus considering the complexity of this event and generating evidence to guide decision-making for comprehensive trauma care.^11^ It was found that the median disability of participants, evaluated after the year of the trauma, was 14.93 (3.84, 97.61), of which 73.15% presented some level of disability, being higher than the one reported in Colombia for the general population according to data from the 2018 Census (7.1%).^38^

When comparing with international reports evaluating post-trauma disability, it was found that the frequency of disability in the subjects studied was higher than reported in European studies in general (30.4%),^39^, ^40^, ^41^ in Asian (14.5%–50%),^42^ in the United States (22%–62%),^31^^,^^43^^,^^44^ and in New Zealand (53.6%),^18^ while the median disability was lower than those reported in Norway (28.1%)^45^ and similar to Australia (14.4%).^46^ No studies with these characteristics were found in Latin America. Among the literature, 2 studies focused exclusively on traffic events.^3^^,^^7^ These and combined with the differences in follow-up periods and sample sizes account for the differences between previous studies and the present study. The findings presented here show that the functional limitations resulting from road traffic injury are long-lasting, persisting beyond the first year after the event.

Following the causal pathways investigated, being a woman was the sociodemographic factor most strongly associated with higher disability scores. When comparing this finding with the literature, it was found that in studies from Norway,^45^ New Zealand,^47^ Australia,^48^ and the United States,^49^ being a woman has been reported as a factor associated with definitive disability following trauma, while in the studies of Iran,^14^ Spain,^7^ Greece,^3^ Italy,^3^ and Germany,^3^ it showed no sex-related relationship or a protective relationship. Potential explanations include the higher prevalence of mental health conditions among women, such as depressive symptoms and anxiety. These conditions may impair the ability to derive enjoyment and disrupt the maintenance of habits and routines, which are essential for performance in the family and community.^50^^,^^51^ Other aspects, such as economic dependence and social isolation, will limit activities and restrict participation in society, which could also explain the greater disability in women in the present study.^52^^,^^53^ However, the results showed that the clinical factors causing disability were the ISS and hospitalization in inpatient and emergency rooms. Regarding the ISS, evidence from other countries demonstrates a direct correlation between injury severity and functional impairment after a traumatic event, which aligns with the findings reported in the existing literature.^3^^,^^14^^,^^16^^,^^50^^,^^54^^,^^55^ Changes in the different structures and/or organs, as reflected by severity indexes, result in greater compromise and ultimate definitive impairment. The relationship is reflected in the consistent correlation between the increase in index score and greater disability.

Also, in the subjects studied, the days of hospitalization in inpatient and emergency rooms explain the disability. Coinciding with studies in similar populations,^49^ the various factors during the hospitalization period, such as exposure to various types of medications, prolonged bed rest, and limited interaction with the external environment, contribute to the functional compromise after patients are discharged from the hospital.^55^^,^^56^

Finally, disability decreases progressively over time following the initial event, a trend consistent with long-term follow-up (>1 year) studies from Norway,^16^ Hong Kong,^56^ and Australia,^57^ which is attributed to sustained environment interaction and activity adaptation, maintaining in the long term. The literature suggests that during the first year after the occurrence of event, the main changes occur, such as the return to education and employment activities, among others, which increase the functionality of the affected population and are maintained after 1 year, increasing functionality to a lesser degree after that time.^16^^,^^48^^,^^57^^,^^58^^,^^59^

On the other hand, when evaluating the relationship between factors related to access to rehabilitation services and disability, 4 of these formed the final structural model of causal pathways of the disability score: not receiving rehabilitation, hospital readmission, duration of rehabilitation, and presence of a caregiver.^59^ Through statistical regression analysis in the studies of Sirois et al.^60^ in Canada, Soberg et al.^16^ in Norway, Derret et al.,^12^ Langley et al.,^18^ and Maclenan et al.^53^ in New Zealand, the rehabilitation services were associated with functional impairment in the population surviving any type of trauma, which was similar to the present research.

Participants needing a caregiver for daily activities demonstrated significantly greater disability levels than those functioning independently. This finding coincides with research conducted on people with severe mental disorders, in events such as stroke and neurological disorders,^61^, ^62^, ^63^ which reported the need for a caregiver as a factor directly associated with disability in the studied population. Additionally, 13% (*n* = 34) of the subjects in this study reported having a caregiver to assist with their daily activities, though these people were affected by less severe trauma. According to the literature, the amount and quality of care depend on factors such as time, the relationship between the affected person and the caregiver, the characteristics of the injury, and the caregiver's level of training.^64^^,^^65^ The relationship between requiring a caregiver and the level of disability found here could be explained by a lack of caregiver training to accurately define the activities in which the affected person really requires their support, thus enhancing their skills.^66^ Under these conditions, having a caregiver may lead to an increase in disability due to high levels of dependence or overprotection,^67^ which highlights the importance of early rehabilitation and incorporation of families and caregivers. Another possible explanation for this finding is the presence of a confounding effect by indication (of treatment or therapy), given the initial severity of the trauma.^68^

The results demonstrate no evidence for the use of rehabilitation service mediating the causal pathway of disability in the study. However, hospitalization in ICU and caregiver were found as significant mediators between ISS and disability. The mediating effect increases disability independently, beyond other possible evaluated mediators. No studies were found that evaluated the mediation of rehabilitation services in the prediction of medium-term disability, but other mediating effects, such as physical activity, barriers to participation, and psychological aspects, have been evaluated to explain disability in other populations.^69^, ^70^, ^71^, ^72^ The mediation of a caregiver in the relationship between ICU stay and disability has different effects on mental health, such as anxiety, depression, post-traumatic stress disorder, etc. The relationship is conceptualized in the literature as post-intensive care syndrome, which influences the level of burden assumed by caregivers and families for caring for trauma patient, and remains after the year of hospital discharge. In addition to the above, the hospital stay in ICU may have an effect that has been documented for other illnesses.^72^^,^^73^

There were still some limitations in the study. Although validated instruments (WHODAS 2.0) were used in the telephone-based survey and were evaluated for their applicability, measurements with this type of instrument may be affected by reporting biases. Particularly, the measurement through the telephone-based survey may be affected by recall bias, in such a way that people with greater severity of injury may remember more accurately aspects of the rehabilitation process in comparison with people with less severity of injury. Socio-demographic comparisons between participants and non-participants revealed sex differences, with women demonstrating higher participation rates. However, the distribution in the study sample is similar to that of the subjects registered in institutional databases. Given few studies on disability among road traffic survivors in Latin America, the results in this study provide a contribution to understanding functional impairment in this population in the southwest Colombia. The study's commitment to addressing disability from a biopsychosocial approach—considering the interrelationships between sociodemographic and clinical factors, as well as access to rehabilitation services—represents progress in the effort to understand traffic-related disability in all its complexity.

In conclusion, disability a year after road traffic injury is not solely determined by the biological conditions of the individual. Instead, it results from complex interrelationships between the individual conditions and their environment, particularly the access to rehabilitation services. Although the use of services did not mediate the relationship in explaining disability, the results show multiple interrelated factors. Sociodemography, clinic, and access-to-rehabilitation characteristics are closely interconnected. Individual aspects, such as sex and injury severity, interact with environmental factors like hospital stay. These, in turn, influence dynamics in the home environment, such as the relationship with the caregiver and the likelihood of hospital readmission. These interrelationships persist beyond the year of occurrence of the injury and influence their functional improvement. The caregiver's mediating effect was found in the relationship between clinical conditions and disability, which highlights the importance of rehabilitation service during the hospital stay and the inclusion of families and caregivers. Future research should further explore this topic.

## CRediT authorship contribution statement

**Lina Marcela Sandoval:** Writing – original draft, Supervision, Project administration, Visualization, Methodology, Funding acquisition, Conceptualization, Writing – review & editing, Validation, Resources, Investigation, Formal analysis, Data curation. **Andrés Fandiño-Losada:** Writing – original draft, Methodology, Conceptualization, Writing – review & editing, Validation, Formal analysis. **Elvis Siprian Castro-Alzate:** Project administration, Investigation, Writing – review & editing, Resources, Conceptualization, Supervision, Writing – original draft, Methodology, Funding acquisition. **Claudio Bustos:** Writing – review & editing, Validation, Formal analysis, Visualization, Investigation, Data curation, Writing – original draft. **Alberto Federico García:** Writing – original draft, Project administration, Resources, Conceptualization, Writing – review & editing, Visualization, Funding acquisition, Validation, Investigation. **Adrián David Fernández:** Writing – original draft, Supervision, Project administration, Writing – review & editing, Resources, Visualization.

## Ethical statement

This study was conducted in accordance with the ethical principles outlined in the 1964 Declaration of Helsinki and its later amendments, as well as the International Ethical Guidelines for Health-related Research Involving Human issued by the Council for International Organizations of Medical Sciences (CIOMS) in collaboration with the World Health Organization (WHO).

The research was approved by the Human Ethics Committee of the Universidad del Valle according to records id 015-020 and by the Ethics Committee of the participating institutions according to records of 2020 with the id 049 from Hospital Universitario del Valle, 000120 from Clinica Imbanaco, and 386 from Fundación Valle del Lili. All participants provided informed consent prior to participation. Data were anonymized to ensure privacy and confidentiality. Participants were no exposed to any physical, psychological, or legal risk.

## Funding

This study was funded by the Vice-rector's office of Research at 10.13039/501100007329Universidad del Valle, through the call 123 of 2020, internal code 1905.

## Declaration of competing interest

The authors declare that they have no conflict of interest.

## References

[1] (2018). GBD 2017 disease and injury Incidence and Prevalence Collaborators. Global, regional, and national incidence, prevalence, and years lived with disability for 354 diseases and injuries for 195 countries and territories, 1990–2017: a systematic analysis for the global Burden of Disease Study 2017. Lancet.

[2] Barth J., Kopfmann S., Nyberg E. (2005). Posttraumatic stress disorders and extent of psychosocial impairments five years after a traffic accident. Psycho Soc Med.

[3] Papadakaki M., Ferraro O.E., Orsi C. (2017). Psychological distress and physical disability in patients sustaining severe injuries in road traffic crashes: results from a one-year cohort study from three European countries. Injury.

[4] Alemany R., Ayuso M., Guillén M. (2013). Impact of road traffic injuries on disability rates and long-term care costs in Spain. Accid Anal Prev.

[5] Barnes J., Thomas P. (2006). Quality of life outcomes in a hospitalized sample of road users involved in crashes. Annu Proc Assoc Adv Automot Med.

[6] Berecki-Gisolf J., Collie A., McClure R. (2013). Work disability after road traffic injury in a mixed population with and without hospitalisation. Accid Anal Prev.

[7] Palmera-Suárez R., López-Cuadrado T., Brockhaus S. (2016). Severity of disability related to road traffic crashes in the Spanish adult population. Accid Anal Prev.

[8] Martínez-Ruíz D.M., Fandiño-Losada A., Ponce de Leon A. (2019). Impact evaluation of camera enforcement for traffic violations in Cali, Colombia, 2008-2014. Accid Anal Prev.

[9] De Los Ríos-P Erez A., García A., Cuello L. (2022). Performance of the Paediatric Trauma Score on survival prediction of injured children at a major trauma centre: a retrospective Colombian cohort, 2011−2019. Lancet Reg Health Am.

[10] Instituto Nacional de Medicina Legal y Ciencias Forenses (2020). http://www.medicinalegal.gov.co/documents/20143/787115/Forensis_2020.pdf.

[11] World Health Organization (2001).

[12] Maclennan B., Wyeth E., Hokowhitu B. (2013). Injury severity and 3-month outcomes among Maori: results from a New Zealand prospective cohort study. N Z Med J.

[13] Palmera-Suárez R., López-Cuadrado T., Fernández-Cuenca R. (2018). Inequalities in the risk of disability due to traffic injuries in the Spanish adult population, 2009-2010. Injury.

[14] Abedzadeh-Kalahroudi M., Razi E., Sehat M. (2015). Measurement of disability and Its predictors among trauma patients: a Follow-up Study. Arch Trauma Res.

[15] Soberg H.L., Finset A., Bautz-Holter E. (2007). Return to work after severe multiple injuries: a multidimensional approach on status 1 and 2 years postinjury. J Trauma.

[16] Soberg H.L., Finset A., Roise O. (2012). The trajectory of physical and mental health from injury to 5 years after multiple trauma: a prospective, longitudinal cohort study. Arch Phys Med Rehabil.

[17] Vásquez-Barquero J.L., Castanedo S.H., Bourgon E.V. (2006). Versión Española Del World Health Organization Disability Assessment Schedule II (WHO-DAS II). Madrid: Ministerio De Trabajo Y Asuntos Sociales.

[18] Derrett S., Samaranayaka A., Wilson S. (2012). Prevalence and predictors of sub-acute phase disability after injury among hospitalised and non-hospitalised groups: a longitudinal cohort study. PLoS One.

[19] Szklo M., Jiméz P., Nieto F.J. (2003). Epidemiología intermedia: conceptos y aplicaciones. Madrid: Ediciones Díaz de Santos. Capítulo.

[20] Baker S.P., O'Neill B., Haddon W. (1974). The injury severity score: a method for describing patients with multiple injuries and evaluating emergency care. J Trauma.

[21] Ahrens W., Pigeot I. (2014).

[22] Ustün T.B., Chatterji S., Kostanjsek N. (2010). Developing the world Health Organization disability assessment schedule 2.0. Bull World Health Organ.

[23] Abedzadeh-Kalahroudi M., Razi E., Sehat M. (2016). Psychometric properties of the world health organization disability assessment schedule II -12 Item (WHODAS II) in trauma patients. Injury.

[24] Domingues D., Alvarelhão J.J., Cerqueira M. (2021).

[25] Hair J.F., Black W.C., Babin B.J. (2009).

[26] Byrne B.M. (2012).

[27] Javadizadeh B. (2020). Mplus 8-4: a software review. J Market Anal.

[28] Dempster A.P., Laird N.M., Rubin D.B. (1977). Maximum likelihood from incomplete data via the EM algorithm. J Roy Stat Soc B.

[29] Kaplan D. (2000).

[30] Ullman J.B., Bentler P.M., Tabachnick B.G., Fidell L.S. (2001). Using Multivariate Statistics. Needham Heights.

[31] MacKinnon D. (2012).

[32] Haider A.H., Herrera-Escobar J.P., Al Rafai SS. (2020). Factors associated with long-term outcomes after injury: results of the functional outcomes and recovery after trauma emergencies (FORTE) multicenter cohort Study. Ann Surg.

[33] Herrera-Escobar J.P., Seshadri A.J., Rivero R. (2019). Lower education and income predict worse long-term outcomes after injury. J Trauma Acute Care Surg.

[34] Ramachandran A., Ranjit A., Zogg C.K. (2017). Comparison of epidemiology of the injuries and outcomes in two first-level trauma centers in Colombia using the Pan-American trauma registry System. World J Surg.

[35] Harris I.A., Young J.M., Rae H. (2008). Predictors of general health after major trauma. J Trauma.

[36] Gopinath B., Jagnoor J., Harris I.A. (2015). Prognostic indicators of social outcomes in persons who sustained an injury in a road traffic crash. Injury.

[37] Paiva L., Monteiro D.A., Pompeo D.A. (2015). Readmissions due to traffic accidents at a general hospital. Rev Lat Am Enfermagem.

[38] Departamento Administrativo Nacional de Estadística (DANE) (2025).

[39] Papadakaki M., Ferraro O.E., Orsi C. (2017). Psychological distress and physical disability in patients sustaining severe injuries in road traffic crashes: results from a one-year cohort study from three European countries. Injury.

[40] Barnes J., Thomas P. (2006). Quality of life outcomes in a hospitalized sample of road users involved in crashes. Annu Proc Assoc Adv Automot Med.

[41] Berecki-Gisolf J., Collie A., McClure R. (2013). Work disability after road traffic injury in a mixed population with and without hospitalisation. Accid Anal Prev.

[42] Rainer T.H., Hung K., Yeung J. (2019). Trajectory of functional outcome and health status after moderate-to-major trauma in Hong Kong: a prospective 5 year cohort study. Injury.

[43] Abedzadeh-Kalahroudi M., Razi E., Sehat M. (2018). The relationship between socioeconomic status and trauma outcomes. J Public Health.

[44] Alghnam S., Wegener S.T., Bhalla K. (2015). Long-term outcomes of individuals injured in motor vehicle crashes: a population-based study. Injury.

[45] Soberg H.L., Bautz-Holter E., Roise O. (2007). Long-term multidimensional functional consequences of severe multiple injuries two years after trauma: a prospective longitudinal cohort study. J Trauma.

[46] Hung K., Kifley A., Brown K. (2021). Impacts of injury severity on long-term outcomes following motor vehicle crashes. BMC Public Health.

[47] Fitzharris M., Fildes B., Charlton J. (2007). General health status and functional disability following injury in traffic crashes. Traffic Inj Prev.

[48] Gabbe B.J., Simpson P.M., Cameron P.A. (2017). Long-term health status and trajectories of seriously injured patients: a population-based longitudinal study. PLoS Med.

[49] Haider A.H., Herrera-Escobar J.P., Al Rafai SS. (2020). Factors associated with long-term outcomes after injury: results of the functional outcomes and recovery after trauma emergencies (FORTE) multicenter cohort Study. Ann Surg.

[50] Tenorio-Martínez R., del Carmen Lara-Muñoz M., Medina-Mora M.E. (2009). Measurement of problems in activities and participation in patients with anxiety, depression and schizophrenia using the ICF checklist. Soc Psychiatry Psychiatr Epidemiol.

[51] Hudson J.L., Bower P., Archer J. (2016). Does collaborative care improve social functioning in adults with depression? The application of the WHO ICF framework and meta-analysis of outcomes. J Affect Disord.

[52] Kellezi B., Dhiman P., Coupland C. (2022). Mental health and other factors associated with work productivity after injury in the UK: Multicentre cohort study. Inj Prev.

[53] Langley J., Derrett S., Davie G. (2011). A cohort study of short-term functional outcomes following injury: the role of pre-injury socio-demographic and health characteristics, injury and injury-related healthcare. Health Qual Life Outcome.

[54] Lugo L.H., García H.I., Cano B.C. (2013). Multicentric study of epidemiological and clinical characteristics of persons injured in motor vehicle accidents in Medellín, Colombia, 2009-2010. Colomb Méd.

[55] Giral M., Boussat B., Lombard F. (2018). Looking at hospitalized persons throughout the prism of the handicap. Ann Phys Rehabil Med.

[56] Hajjioui A., Fourtassi M., Nejjari C. (2015). Prevalence of disability and rehabilitation needs amongst adult hospitalized patients in a Moroccan university hospital. J Rehabil Med.

[57] Rainer T.H., Hung K., Yeung J. (2019). Trajectory of functional outcome and health status after moderate-to-major trauma in Hong Kong: a prospective 5 year cohort study. Injury.

[58] Henao-Lema C.P., Pérez-Parra J.E. (2011). Modelo predictivo del grado de discapacidad en adultos con lesión medular: resultados desde el WHO-DAS II. Rev Cien Salud.

[59] Krause J.S., Coker J.L. (2006). Aging after spinal cord injury: a 30-year longitudinal study. J Spinal Cord Med.

[60] Sirois M.J., Dionne C.E., Lavoie A. (2009). Regional differences in rehabilitation needs, rehabilitation access, and physical outcomes among multiple trauma survivors. Am J Phys Med Rehabil.

[61] Camargo-Rojas D., Castro-Alzate E., Hernández-Romero H. (2015). Conocimientos, actitudes y prácticas de cuidadores de personas con discapacidad, en procesos de inclusión social en el municipio Madrid, Cundinamarca, Colombia. Ciencias de la Salud.

[62] Castro-Alzate E.S., Cardona-Marín L.M., López R.P. (2021). Modelo explicativo de discapacidad en población con trastornos mentales graves: un estudio multicéntrico en tres países de Sudamérica. Rev Cien Salud.

[63] Szkultecka-Dębek M., Miernik K., Stelmachowski J. (2016). Schizophrenia causes significant burden to patients' and caregivers' lives. Psychiatr Danub.

[64] Rodríguez-González A.M., Rodríguez-Míguez E., Duarte-Pérez A. (2017). Cross-sectional study of informal caregiver burden and the determinants related to the care of dependent persons. Aten Primaria.

[65] Pérez Peñaranda A. (2015). Tesis De Maestría.

[66] Bejarano Espitia, Carolina Diana, Diana Carolina Espitia Bejarano Resumen Abstrac. :1–38 (2015).

[67] Toboso-Martin M., Arnau M.S. (2008). La discapacidad dentro del enfoque de capacidades y funcionamientos de Amartya Sen. Revista Iberoamericana de Filosofía, Política y Humanidades.

[68] Bosco J.L., Silliman R.A., Thwin S.S. (2010). A most stubborn bias: no adjustment method fully resolves confounding by indication in observational studies. J Clin Epidemiol.

[69] Aitken Z., Bishop G.M., Disney G. (2022). Disability-related inequalities in health and well-being are mediated by barriers to participation faced by people with disability. A causal mediation analysis. Soc Sci Med.

[70] Marshall P.W.M., Schabrun S., Knox M.F. (2017). Physical activity and the mediating effect of fear, depression, anxiety, and catastrophizing on pain related disability in people with chronic low back pain. PLoS One.

[71] Badu E., O'Brien A.P., Mitchell R. (2020). Mediation and moderation effects of health system structure and process on the quality of mental health services in Ghana - structural equation modelling. PLoS One.

[72] Torres J., Carvalho D., Molinos E. (2017). The impact of the patient post-intensive care syndrome components upon caregiver burden. Med Intensiva.

[73] Inoue S., Hatakeyama J., Kondo Y. (2019). Post-intensive care syndrome: its pathophysiology, prevention, and future directions. Acute Med Surg.

